# Prevalence and Risk Factors for Iron Deficiency Anemia among Children under Five and Women of Reproductive Age in Pakistan: Findings from the National Nutrition Survey 2018

**DOI:** 10.3390/nu15153361

**Published:** 2023-07-28

**Authors:** Atif Habib, Sumra Kureishy, Sajid Soofi, Imtiaz Hussain, Arjumand Rizvi, Imran Ahmed, Khawaja Masuood Ahmed, Abdul Baseer Khan Achakzai, Zulfiqar A. Bhutta

**Affiliations:** 1 Center of Excellence in Women & Child Health, The Aga Khan University, Karachi 74800, Pakistan; 2 Ministry of Health Services Regulation & Coordination, Islamabad 44020, Pakistanachakzaibk@gmail.com (A.B.K.A.); 3 Lawson Centre for Nutrition, University of Toronto, Toronto, ON M5S 1A8, Canada; 4 Centre for Global Child Health, Hospital for Sick Children, Toronto, ON M5G 1X8, Canada

**Keywords:** iron-deficiency anemia, risk factors, children under five, women of reproductive age, Pakistan

## Abstract

Introduction: Anemia remains a global public health problem, especially in developing countries. It affects primarily children under five (CU5), women of reproductive age (WRA), and pregnant women due to their higher need for iron. The most common form of anemia is iron-deficiency anemia (IDA). IDA is estimated to cause half of all anemia cases and one million deaths per year worldwide. However, there remains a lack of well-documented and biochemically assessed prevalence of IDA based on the representative population-based samples globally and regionally. In this study, we aimed to assess the National Nutrition Survey (NNS) 2018 to identify the prevalence and risk factors of IDA in Pakistani CU5 and WRA. Methods: Secondary analysis was conducted on the NNS 2018, a cross-sectional survey, which collected data on dietary practices, malnutrition, and food insecurity. Anemia was defined as hemoglobin levels < 11.0 g/dL in children and 12.0 g/dL in women. IDA was defined as low hemoglobin and low ferritin (<12 ng/mL) levels, adjusted for inflammation using AGP and CRP biomarkers in CU5 and WRA. Univariate and multivariable logistic regressions were conducted using Stata statistical software (version 16). We also compared the IDA rates of NNS 2018 and 2011. Results: A total of 17,814 CU5 and 22,114 WRA were included in the analysis. Of the CU5, 28.9% had IDA, while 18.4% of WRA reported to experience IDA. Among the CU5, IDA was most prevalent among male children aged 6–23 months living in rural areas and with the presence of diarrhea and fevers in the last 2 weeks. Children whose mothers had no education, were aged 20–34 years, and employed, had a higher prevalence of IDA. Married WRA, who are employed, living in rural areas, and with no education, had a higher prevalence of IDA. In the multivariable logistic regression, children aged 6–23 months (AOR = 1.19, 95% CI [1.08–1.33], *p* < 0.001) and with the presence of diarrhea in the last 2 weeks (AOR = 1.32, 95% CI [1.13–1.54], *p* < 0.001) or fever (AOR = 1.16, 95% CI [1.02–1.32], *p* = 0.02) had higher odds of IDA. At the household level, the odds of IDA among CU5 were higher in the poorest households (AOR = 1.27, 95% CI [1.08–1.50], *p* = 0.005), with ≥5 CU5 (AOR = 1.99, 95% CI [1.28–3.11], *p* = 0.002), and with no access to improved sanitation facilities (AOR = 1.17, 95% CI [1.02–1.34], *p* = 0.026). For WRA, the multivariable logistic regression found that the odds of IDA were higher among women with vitamin A deficiency (Severe: AOR = 1.26, 95% CI [1.05–1.52], *p* = 0.013; Mild: AOR = 1.36, 95% CI [1.23–1.51], *p* < 0.001), zinc deficiency (AOR = 1.42, 95% CI [1.28–1.57], *p* < 0.001), no education (AOR = 1.53, 95% CI [1.30–1.81], *p* < 0.001), and from severely food insecure households (AOR = 1.20, 95% CI [1.07–1.34], *p* = 0.001). The odds of IDA were lower among women whose body mass index was overweight (AOR = 0.77, 95% CI [0.69–0.86], *p* < 0.001) or obese (AOR = 0.71, 95% CI [0.62–0.81], *p* < 0.001). Conclusions: The child’s age, presence of diarrhea or fever, place of residence, household size, wealth status, and access to sanitation facilities were significantly associated with IDA among CU5 in Pakistan. For WRA, education, body mass index, vitamin A and zinc status, household food security status, wealth status, and access to sanitation facilities were significantly associated with IDA. Large, well-established, government-funded programmes focused on micronutrient supplementation, food fortification, the diversification of food supplies, and the treatment and prevention of infectious and parasitic diseases are needed to prevent IDA and all forms of anemia among children and women in Pakistan.

## 1. Introduction

Anemia remains a global public health problem, especially in developing countries [1,2,3]. It is characterized by low hemoglobin (Hb) concentrations (<11.0 g/dL), which indicate limited or abnormal red blood cells with decreased capacities to meet the body’s oxygen requirements [4]. This increases the risk of maternal and child morbidity and mortality, specifically neural tube defects, low birth weight, premature birth, early fetal loss, impaired physical and cognitive child development, poor school performance, and limited adult productivity [5]. Anemia can also occur due to other micronutrient deficiencies (vitamin B12 and folate), poor nutritional intake, hemoglobinopathies, and the presence of infectious diseases (malaria, helminth infections, and other infections such as tuberculosis and HIV/AIDS) [3]. It affects primarily children under five (CU5), women of reproductive age (WRA), and pregnant women due to their higher need for iron. An estimated 39.8% of CU5, 29.9% of WRA, and 36.5% of pregnant women are anemic worldwide [6,7,8]. In South Asia, the prevalence of anemia is 52% among CU5 and 49% among WRA [6,7]. Moreover, when focusing on the seven countries within the region, Pakistan has the second-highest prevalence (53%) of anemia among CU5 and the fourth-highest prevalence (41.3%) of anemia among WRA [9,10,11,12,13,14,15,16,17,18]. According to WHO guidelines, anemia is deemed a public health problem when the prevalence is ≥5%, with its public health significance being mild between 5 and 19%, moderate between 20 and 29%, and severe ≥40% in any group [19]. The most common form of anemia is iron-deficiency anemia (IDA) [3,19]. Previous studies have estimated that iron deficiency is responsible for half of all anemia cases and one million deaths per year worldwide [3,20,21]. It is also estimated to cause an annual loss of 4.05% of GDP or USD 16.78 per capita due to impaired physical and cognitive productivity worldwide [20]. However, there is a lack of well-documented and biochemically assessed prevalence of IDA based on the representative global and regional population-based samples, as its confirmation requires two biochemical analyses indicating low hemoglobin (<11.0 g/dL in CU5; <12.0 g/dL in WRA) and low ferritin (<12 ng/mL) levels [3]. Ferritin levels can be affected by inflammation; therefore, it should be adjusted for inflammation using C-reactive protein and alpha-acid glycoprotein.

Over the past 20 years, the prevalence of anemia in CU5 (53.7% in 2018) and WRA (42.7% in 2018) has remained a severe public health problem in Pakistan [10]. There has also been very little improvement made in the prevalence of IDA among Pakistani CU5 and WRA. A minor 7% reduction has been observed in the prevalence of IDA in both CU5 (from 35.6% to 28.6%) and WRA (from 25.5% to 18.2%) between 2001 and 2018. Currently, there exist no national-level, government-funded programmes aimed at reducing the prevalence of IDA or other forms of anemia among young children and WRA. The government does, however, provide funding for the provision of iron-folic acid supplementation to pregnant women through the Lady Health Worker Programme [22,23]. Moreover, to address micronutrient deficiencies among the general population, the country has implemented a donor-funded five-year large-scale food fortification programme for wheat flour and edible oil [24,25,26]. This study aims to assess the National Nutrition Survey 2018, the most recent nationally representative data, to determine the prevalence and risk factors of IDA in Pakistan. These results may help prioritize key measures to reduce IDA and other micronutrient deficiencies amongst children and WRA.

## 2. Materials and Methods

The National Nutrition Survey 2018, a cross-sectional survey, collected quantitative and qualitative data at the household level across the country [10]. A two-stage stratified sampling design was used, where the primary sampling units (PSUs) consisted of one village/city or part of a village/city containing an average of 200–250 households. These households were treated as secondary sampling units (SSU). The district-level representative sample size was calculated using the most recent prevalence of wasting, stunting, and micronutrient deficiencies in children, adolescents, and women. A total of 115,600 households were sampled to collect data on malnutrition, dietary practices, and food insecurity from all districts of Azad Jammu and Kashmir (AJK), Balochistan, Gilgit-Baltistan (GB), Islamabad Capital Territory (ICT), Khyber Pakhtunkhwa (including erstwhile Federally Administered Tribal Areas [FATA]), Punjab, and Sindh. Children under five (0–59 months), school-aged children (6–12 years), adolescents (10–19 years), and women of reproductive age (15–49 years) were the main target population of the survey. Additional details on the national survey design, methodology, and results are presented elsewhere [10].

Secondary data from 17,814 CU5 (3471 children aged 6–23 months and 14,343 children aged 24–59 months) and 22,114 WRA was used to identify the prevalence and risk factors of IDA by maternal age, education, and employment; child gender, age, and presence of disease; women’s age, education, employment, and presence of disease; household socioeconomic status and place of residence. Univariate logistic regression was conducted to identify factors associated with IDA in CU5 and WRA. All data analyses were conducted in STATA version 16. Since IDA was the main outcome measure, women and children with results available for both hemoglobin and ferritin were included in the analysis. The frequencies, along with weighted percentages, were reported for selected predictors. The analysis started with simple univariate analysis followed by multivariate logistic regression. Unadjusted odds ratios with their 95% CIs were reported for the bivariate analysis. Variables significant at *p* < 0.25 were considered for inclusion in the multivariate model. Covariates that were not significant at the multivariate level were dropped from the model after careful assessment of confounding. The final model was selected based on theoretical and statistical significance of predictors. The Type 1 error was set at 0.05. The model estimates are presented with the adjusted odds ratios (AOR) and 95% CI.

The presence of anemia and IDA was assessed in CU5 and WRA via blood samples and an on-the-spot hemoglobin test using a HemoCue 301 machine. Anemia was defined as hemoglobin levels <11.0 g/dL in children and 12.0 g/dL in women [27]. IDA was defined as low hemoglobin and low ferritin (<12 ng/mL) levels, adjusted for inflammation using AGP and CRP biomarkers in CU5 and WRA. To evaluate CRP and AGP, micronutrient assays were conducted to measure plasma C-reactive protein and alpha-1-acid glycoprotein. Cobas c311, an automated chemistry analyzer (Roche, Basel, Switzerland), was used to quantify these proteins [28]. The analyzer performed an in vitro particle-enhanced immunoturbidimetric test that utilized latex to enhance the interaction between antigens and antibodies based on the immunological agglutination principle. The resulting complexes were then read turbidimetrically at specific wavelengths 660/340 nm for AGP and 546 nm for CRP. To ensure quality control, two levels of internal QC materials, Precicontrol Clinchem Multi levels 1 and 2 (Roche, Basel, Switzerland), were used at least once every twenty-four hours whenever a new assay kit or reagent was introduced or after performing a fresh calibration [29]. Any result falling outside the typical reference range was identified and rechecked immediately. The CRP values were reported to be the nearest tenth in mg/dL, with the AMR (Analytical Measurement Range) for the CRP assay being 0.1–0.25 mg/dL. The normal range for CRP was defined as 0–0.5 mg/dL. While for the AGP assay, the Roche/Hitachi Cobas c systems automatically calculated the concentration of the analyte in each sample and reported it in mg/dL. The AMR for the AGP assay was 10–400 mg/dL, where individuals with values greater than or equal to 100 mg/dL were considered to have elevated levels of AGP.

To determine the plasma content of vitamin A (retinol), a reverse-phase high-pressure liquid chromatographic (HPLC) technique was employed. This analytical method helps separate and quantify different components in a mixture based on their chemical properties and retention times. The retinol levels were quantified using an Agilent 1200 series HPLC analyzer (Agilent, Santa Clara, CA, USA) with UV/Visible detection. To separate the components, a ZORBAX C-18 column measuring 4.6 × 150 mm with a particle size of 5 µm was utilized, and the analyte’s concentration was measured at 325 nm. The mobile phase consisted of 100% methanol, maintained at a constant composition throughout the analysis. In order to correct for variable recovery, retinyl acetate was employed as an internal standard.

The measurement of plasma levels of 25-hydroxy vitamin D was performed using an automatic immunoanalyzer called Diasorin Liaison (Saluggia, VC, Italy). The technique employed for this analysis was a direct competitive chemiluminescence immunoassay (CLIA), which utilizes the principle of competition between the analyte (25-hydroxy vitamin D) and a labelled counterpart for binding to a specific antibody. Analytical Measuring Range (AMR) for the Liaison XL 25 OH Vitamin D assay is 4 to 130 ng/mL. Values < 20 were considered to be associated with vitamin D deficiency [30]. Plasma samples were analyzed for zinc estimation using an atomic absorption spectrophotometer, model ICE 3000 series (Thermofisher, Waltham, MA, USA). This technique relies on the fact that metals in their ground state absorb light at specific wavelengths. To achieve this, metal ions in a solution were converted to an atomic state through a flame. By supplying light at the appropriate wavelength (319 nm), the amount of absorbed light was measured against a standard curve. A flame with a flow rate of 0.9 to 1.2 L per minute (Air/acetylene) was used, providing a temperature of 2500 °C required for the atomization of gaseous zinc molecules within the flame. To ensure quality control, we employed serum controls from “UTAK Laboratories, Inc., Valencia, CA, USA” These controls were diluted with a specified volume of de-ionized water and remained stable for 30 days after dilution when stored between 2 and 8 °C. The controls were treated in the same manner as the analytical samples, meaning they were also diluted at a ratio of 1:10 with de-ionized water and then aspirated into the flame. A normal level of zinc in blood serum/plasma was considered to be less than 160 micrograms/dL [31]. A countrywide network of laboratories and collection centers was used to transport blood samples, maintain a cold chain, and ensure viability. The samples were analyzed at the Aga Khan University (AKU), Karachi, Sindh. The ethical clearance for the National Nutrition Survey and secondary analysis was obtained from Aga Khan University’s Ethical Review Committee (ERC) and the National Bioethics Committee (NBC). All participants provided informed consent before survey enrolment. The secondary data analysis was approved by the ERC at AKU. Informed consent was obtained from all participants before the recruitment in this study, data collection, hematological investigations, and anthropometric measurements. The consent forms outlined the objectives, measurements, and process of confidentiality of this study.

## 3. Results

### 3.1. Child, Maternal, and Household Characteristics

Out of the 17,814 CU5, 51.0% were male, with the majority aged 24–59 months (80.6%) and living in rural areas (62.1%) (Table 1). The majority of the children CU5 were from Punjab (55.1%) and Sindh (27.2%), while 0.5% were from Gilgit Baltistan. A higher proportion of children had no reported presence of diarrhea (92.4%), acute respiratory infection (98.1%), and fever (86.3%) in the last 2 weeks. The mean Hb level of 10.6 ± 2.5 and ferritin level of 20.2 ± 32.3 was recorded among the CU5 enrolled in this study. Of the CU5, 28.9% were found experiencing IDA. More than ½ of the mothers of the children had no education (54.8%), while a ¼ had completed secondary (12.4%) or higher education (10.4%). Most of these women were aged 20–34 years (71.5%) and were housewives (89.6%). Most households comprised ≤6 family members (57.7%), had <5 children under the age of five (99.1%), and had access to improved drinking water (92.4%) and improved sanitation facilities (84.4%). Furthermore, 60.8% of households reported being food secure, with 19.1% reporting severe food insecurity. A total of 5 out of 100 households (4.9%) reported receiving some form of financial assistance from the government in the last 12 months.

### 3.2. Women and Household Characteristics

Out of the 22,114 WRA, 93.1% were married, with the majority aged 30–49 years (55.1%) and living in rural areas (60.3%) (Table 2). The majority of the WRA were from Punjab (53.7%) and Sindh (28.2%), while 0.5% were from Gilgit Baltistan. The mean Hb level of 12.1 ± 2.1 and ferritin level of 32.5 ± 61.1 was recorded among the WRA enrolled in the study. Of the WRA, 18.4% were found experiencing IDA. More WRA had a normal body mass index (45.4%; BMI 18.5–24.9 kg/m^2^), normal vitamin A (72.9%), and zinc serum levels (77.8%). Additionally, about 79% of WRA were found to be deficient in vitamin D. More than ½ of the WRA had no education (53.7%), while a ¼ had completed secondary (12.9%) or higher education (11.7%). Most of these women were housewives (86.5%), belonging to households with ≤6 family members (58.0%) and had access to improved drinking water (92.4%) and improved sanitation facilities (84.8%). Furthermore, 60.3% of households with the WRA were food secure, with 19.6% reporting severe food insecurity. A total of 5 out of 100 households (5.2%) reported receiving some form of financial assistance from the government in the last 12 months.

### 3.3. Risk Factors for Iron Deficiency Anemia in Children under Five

In the univariate logistic analysis, the odds of IDA were higher among children under two years of age (OR = 1.179, 95% CI [1.06–1.31], *p* = 0.002) with the presence of diarrhea (OR = 1.42, 95% CI [1.22–1.64], *p* < 0.001) or fever (OR = 1.26, 95% CI [1.12–1.42], *p* < 0.001) in the last 2 weeks compared with children aged 24–59 months with no diarrhea or fever (Table 3). Compared with children whose mothers had higher education, the odds of IDA were higher among children born to mothers with no education (OR = 1.203, 95% CI [1.03–1.40], *p* = 0.017) or primary levels of education (Primary: OR = 1.31, 95% CI [1.09–1.57], *p* = 0.005). Children with employed mothers (OR = 1.04, 95% CI [0.90–1.19], *p* = 0.617) had higher odds of IDA compared with children whose mothers were housewives but not significantly. In addition, the odds of IDA among children decreased with an increase in the mother’s age; however, the odds ratios were not statistically significant for mothers aged 20–34 years (OR =1.173 (95%CI [0.83–1.66], *p* = 0.365) and 35–49 years (OR = 1.00, 95% CI [0.71–1.43], *p* = 0.974), compared to mothers <20 years of age. The odds of IDA in children were higher in households with ≥7 members (OR = 1.11, 95% CI [1.02–1.21], *p* = 0.02), ≥5 CU5 (OR = 1.867, 95% CI [1.21–2.87], *p* = 0.005), and no access to improved sanitation facilities (OR = 1.32, 95% CI [1.18–1.47], *p* < 0.001) compared with households having ≤6 members, <5 CU5, and access to improved sanitation facilities. The odds of IDA increased with the severity of food insecurity experienced by households but was only statistically significant among households experiencing moderate food insecurity (OR = 1.22, 95% CI [1.04–1.43], *p* = 0.013) compared with food secure households. Similarly, the odds of IDA in children increased as the wealth status of households worsened. Across the wealth quintiles, the poorest households (OR = 1.31, 95% CI [1.14–1.51], *p* < 0.001) were most at risk of experiencing IDA in children compared with the richest households. The odds of IDA were lower among children whose households did not receive financial assistance in the last 12 months (OR = 0.66, 95% CI [0.55–0.79], *p* < 0.001) compared with households who received assistance and located in Punjab (OR = 2.49, 95% CI [2.02–3.06], *p* < 0.001), Sindh (OR = 2.77, 95% CI [2.24–3.44], *p* < 0.001), erstwhile FATA (OR = 1.63, 95% CI [1.11–2.41], *p* = 0.014), Balochistan (OR = 1.53, 95% CI [1.19–1.96], *p* < 0.001), Khyber Pakhtunkhwa (OR = 1.43, 95% CI [1.12–1.82], *p* = 0.004), and AJK (OR = 1.31, 95% CI [1.00–1.71], *p* = 0.048) continued to experience higher odds of IDA compared with households located in GB.

In the multivariable logistic regression, children aged 6–23 months (AOR = 1.19, 95% CI [1.08–1.33], *p* < 0.001) with the presence of diarrhea in the last 2 weeks (AOR = 1.32, 95% CI [1.13–1.54], *p* < 0.001) or fever (AOR = 1.16, 95% CI [1.02–1.32], *p* = 0.02) remained at risk of IDA compared with children aged 24–59 months with no diarrhea or fever in the last 2 weeks. The relationship between the risk of IDA among CU5 and maternal age, education, and employment status did not remain apparent, which was initially found to be significant in the univariate analysis. At the household level, the risk of IDA among CU5 remained higher in households with ≥5 CU5 (AOR = 1.99, 95% CI [1.28–3.11], *p* = 0.002), and with no access to improved sanitation facilities (AOR = 1.17, 95% CI [1.02–1.34], *p* = 0.026) compared with households with <5 CU5 and access to improved sanitation facilities. Similarly, the poorest households (AOR = 1.27, 95% CI [1.08–1.50], *p* = 0.005) remained most at risk of experiencing IDA, with the risk gradually decreasing across the wealth quintiles relative to the richest households. Households located in Punjab (AOR = 2.93, 95% CI [2.37–3.63], *p* < 0.001), Sindh (AOR = 2.90, 95% CI [2.33–3.62], *p* < 0.001), erstwhile FATA (AOR = 1.62, 95% CI [1.09–2.41], *p* = 0.017), Balochistan (AOR = 1.58, 95% CI [1.23–2.03], *p* < 0.001), Khyber Pakhtunkhwa (AOR = 1.55, 95% CI [1.22–1.98], *p* < 0.001), and AJK (AOR = 1.44, 95% CI [1.09–1.89], *p* = 0.008) continued to experience higher odds of IDA compared with households located in GB.

### 3.4. Risk Factors for Iron Deficiency Anemia in Women of Reproductive Age

In the univariate logistic analysis, the odds of IDA were higher among women from rural areas (OR = 1.13, 95% CI [1.03–1.24], *p* = 0.008), with the presence of vitamin A deficiency (Severe: OR = 1.36, 95% CI [1.13–1.64], *p* = 0.001; Mild: OR = 1.44, 95% CI [1.31–1.59], *p* < 0.001), zinc deficiency (OR = 1.50, 95% CI [1.36–1.66], *p* < 0.001), and an underweight body mass index (OR = 1.26, 95% CI [1.10–1.44], *p* = 0.001) compared with women from urban areas, with no vitamin A deficiency, zinc deficiency, and an average body mass index (Table 4). Unexpectedly, the risk of IDA among WRA was lower among women with an overweight (OR = 0.71, 95% CI [0.64–0.79], *p* < 0.001) or obese (OR = 0.65, 95% CI [0.56–0.74], *p* < 0.001) body mass index compared with an average body mass index. Compared with women having higher education, the odds of IDA were higher among women with no education (OR = 1.70, 95% CI [1.45–2.00], *p* < 0.001) or lower levels of education (Primary: OR = 1.35, 95% CI [1.10–1.64], *p* = 0.003; Middle: OR = 1.43, 95% CI [1.16–1.76], *p* < 0.001; Secondary: OR = 1.10, 95% CI [0.90–1.35], *p* = 0.337). Employed women (OR = 1.12, 95% CI [0.99–1.27], *p* = 0.058) had higher odds of IDA compared with housewives. The odds of IDA decreased with an increase in the women’s age; however, the odds ratios were not statistically significant for women aged 30–49 years (OR =0.98 (95% CI [0.89–1.07], *p* = 0.668) relative to women 15–29 years of age. Similarly, marital status had no statistically significant association with IDA among women. The odds of IDA were higher in households with no access to improved sanitation facilities (OR = 1.56, 95% CI [1.39–1.73] *p* < 0.001) and were experiencing food insecurity (Severe: OR = 1.46, 95% CI [1.31–1.63], *p* < 0.001; Moderate: OR = 1.32, 95% CI [1.12–1.55], *p* = 0.001; Mild: OR = 1.29, 95% CI [1.12–1.48], *p* < 0.001) compared with households having access to improved sanitation facilities and being food secure. The odds of IDA increased with the severity of food insecurity and poverty experienced by households. The poorest households (OR = 1.75, 95% CI [1.51–2.02], *p* < 0.001) were most at risk of experiencing IDA compared with the richest households. Similar to CU5, the odds of IDA were lower among WRA whose households did not receive financial assistance in the last 12 months (OR = 0.65, 95% CI [0.55–0.78], *p* < 0.001) and were located in Khyber Pakhtunkhwa (OR = 0.461, 95% CI [0.36–0.58], *p* < 0.001), and ICT (OR = 0.62, 95% CI [0.41–0.95], *p* = 0.029) compared with households who received assistance and were located in GB. However, households located in Sindh (OR = 1.39, 95% CI [1.15–1.69], *p* = 0.001) were found to have a higher risk of IDA in WRA compared to GB.

In the multivariable logistic regression, women with the presence of vitamin A deficiency (Severe: AOR = 1.26, 95% CI [1.05–1.52], *p* = 0.013; Mild: AOR = 1.36, 95% CI [1.23–1.51], *p* < 0.001) and zinc deficiency (AOR = 1.42, 95% CI [1.28–1.57], *p* < 0.001) remained at risk of IDA compared with women with no vitamin A deficiency and zinc deficiency. The relationship between the risk of IDA and women’s education remained apparent; however, the odds ratios decreased with no education (AOR = 1.53, 95% CI [1.30–1.81], *p* < 0.001) and lower levels of education (Primary: AOR = 1.26, 95% CI [1.03–1.54], *p* = 0.022; Middle: AOR = 1.39, 95% CI [1.12–1.73], *p* = 0.002). The odds of IDA were lower among women whose body mass index was overweight (AOR = 0.77, 95% CI [0.69–0.86], *p* < 0.001) or obese (AOR = 0.71, 95% CI [0.62–0.81], *p* < 0.001) compared with average body weight women. At the household level, the risk of IDA remained higher in households experiencing severe food insecurity (AOR = 1.20, 95% CI [1.07–1.34], *p* = 0.001) and located in Sindh (AOR = 1.25, 95% CI [1.02–1.52], *p* = 0.028) compared with households experiencing food security and located in GB. Similarly, the risk of IDA remained lower in households located in Khyber Pakhtunkhwa (AOR = 0.74, 95% CI [0.67–0.83], *p* < 0.001) compared to households in GB.

### 3.5. Comparison with Previous National Nutrition Survey 2011

We also compared the data for IDA in the NNS 2018 to the National Nutrition Survey conducted in 2011.

**Figure 1:**
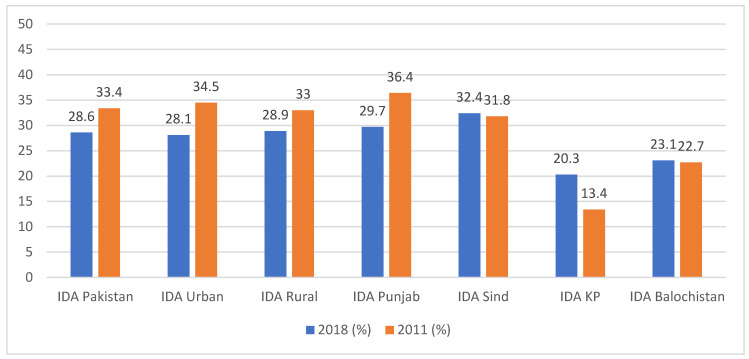
Comparison of IDA among children under five.

The data for children show that the overall IDA prevalence among children in Pakistan was reduced to 28.6% from 33.4%. Similar trends were seen in urban and rural figures. The maximum reduction was found in the Punjab province, whereas for the rest of the provinces, the IDA prevalence was increased (Figure 1).

**Figure 2:**
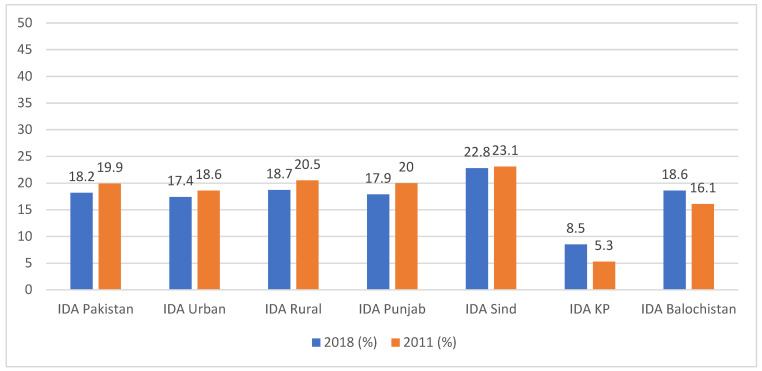
Comparison of IDA among WRA.

Similarly, when looking at the data for WRA, an overall reduction in IDA was observed from 19.9% to 18.2% in Pakistan. Similar trends were seen in urban and rural areas. The provincial data show a reduction in the prevalence of IDA in Punjab and Sind. However, in KP and Baluchistan, the prevalence had gone up (Figure 2).

## 4. Discussion

The prevalence of anemia among CU5 and WRA in Pakistan remains very high compared with global estimates [10]. Although, these prevalence are comparable with regional estimates and surpassed only by India (a country with a large vegetarian population) and Afghanistan (a country experiencing protracted conflict) [5,6,7,8,9,10,11,12,13,14,15,16,17,18]. The high prevalence of IDA among Pakistani children and women found in this study is in line with previous studies [32,33]. However, due to a lack of regional and global estimates determined using representative population-based surveys, the national prevalence of IDA cannot be reflected in detail [18].

This study presents selected risk factors associated with IDA among CU5 and WRA in Pakistan using the National Nutrition Survey 2018. This study shows children’s age, the presence of diarrhea or fever, place of residence, household size, including the number of CU5, household wealth status, and access to sanitation facilities are significantly associated with IDA in CU5. Whilst for WRA, education, BMI, presence of vitamin A and zinc deficiencies, place of residence, household food security status, wealth status, and access to sanitation facilities were significantly associated with IDA. Risk factors associated with greater odds of IDA were children residing in Punjab and Sindh, with ≥5 CU5 experiencing diarrhea in the last 2 weeks and women with no education and deficient in vitamin A and zinc. Women and children from the poorest households, experiencing severe food insecurity and no access to sanitation facilities, had higher odds of IDA. The use of unsafe sanitation facilities can make these women and children more vulnerable to infectious and parasitic diseases and, hence, more likely to experience diarrhea, fever, and IDA [34]. Poverty, on the other hand, limits the ability of CU5 and WRA to access iron-rich nutritious foods. Household food security status was only associated with IDA in WRA; there was no association found between food insecurity and IDA in CU5. With seasonal food insecurity, a common occurrence across the country, women, mothers, and other female caregivers are accustomed to decreasing their portion sizes or skipping meals to ensure adequate food for other households’ members, especially for children [35]. Gender inequality plays a significant role in contributing to anemia in Pakistan. Similar findings related to anemia have been reported within the region [33,36,37,38,39]. In addition, the higher risk of IDA among Punjabi and Sindhi CU5 and Sindhi and Pakhtun WRA may be linked with exposure to province-specific nutrition interventions and dietary habits.

Children aged 6–23 months were more at risk of IDA compared with older children (24–59 months) in this study. This finding is consistent with anemia-related studies conducted in Bangladesh, Bhutan, Nepal, and India [38,40,41,42]. The higher risk of IDA among CU5 may be attributed to the inappropriate initiation of complementary feeding practices. This study also found that women with no education or lower levels of education had a higher risk of IDA compared to women with higher education. Similarly, several studies found that educated women were better informed and able to meet their iron requirements [36,37,38,39,43]. Furthermore, WRA with vitamin A and zinc deficiencies had higher odds of IDA, whereas women with overweight and obese body mass indices were less at risk of IDA compared to non-deficient and average body mass index WRA. Clinical studies have shown that anemia is influenced by vitamin A and zinc deficiency, which leads to impaired hemopoiesis, immunity, iron metabolism, and heme synthesis [44,45,46]. Yet, unlike this study, recent evidence shows that overweight and obese women experience chronic low-grade inflammation and high levels of serum hepcidin, which disturb iron absorption and increase the risk of anemia [47,48,49,50]. There is no clear justification for the lower risk of IDA among overweight and obese women shown in this study. A possible reason behind this protective effect may be higher socioeconomic standing and relative consumption of iron-rich sources.

Policies and programmes supporting micronutrient supplementation, food fortification, diversification of food supplies, and treatment and prevention of infectious and parasitic diseases (malaria, hookworms, etc.) are essential to address IDA (and all forms of anemia) among CU5 and WRA in Pakistan [21]. A multisectoral approach is needed, which also focuses on poverty reduction, female education, dietary diversity, and gender equality. Currently, the country is only providing iron-folic acid supplementation to pregnant women, and vitamin A supplementation and deworming to CU5 via the National Lady Health Worker Programme [22,23]. The programme has and continues to encounter significant financial and human resource issues, often relying on donor funds or in-kind donations of nutrition and health supplies to meet the population’s needs. Supplementation with iron-folic acid for WRA and micronutrient powders for CU5 are usually provided via time-bound, donor-funded, and location-specific projects. The country has also implemented a large-scale food fortification programme (2016–2021) for wheat flour and edible oil with iron, folic acid, vitamin A, vitamin B12, and vitamin D [24,25,26]. This primarily donor-funded programme has built the capacity of millers via training and in-kind provision of premix and micro feeders, strengthened regulatory and quality control infrastructures, and increased the demand and supply of fortified wheat and edible oil. Programme reach has been limited to a third of the country’s population. Through strong political engagement in 2021, the programme has been able to legislate mandatory wheat flour and edible oil fortification in Sindh and Balochistan; while the remaining provinces rely on the 1965 national legislation on mandatory fortification of edible oil. With the programme concluding in 2021, the challenge of sustainability has come to the forefront, especially related to the in-kind provision of premix and micro feeders and the enforcement of legislation and standards. It is evident from our findings that these short-term and funding-restricted programmes and projects have been unable to meet the micronutrient needs of CU5 and WRA.

The major strength of this study is the use of consistent serum hemoglobin and serum ferritin, adjusted for inflammation using AGP and CRP biomarkers, to assess IDA among CU5 and WRA at the population level. Another strength is the use of the most recent, largest nationally representative sample, with a 90% response rate. Some limitations include the use of cross-sectional data, which cannot establish a causal relationship between IDA and the associated risk factors in CU5 and WRA. There have been suggestions that other biomarkers, such as hepcidin [51] and hemojuvelin [52], might improve the assessment of iron deficiency and responsive anemia, although this has not been borne out of trials using a targeted approach to supplementation with hepcidin [53]. Other studies have also suggested doing away with altitude adjustment [54] for anemia, but consensus in this aspect is lacking.

## 5. Conclusions

In conclusion, the study findings reflected that 28.9% of CU5 and 18.4% of WRA had experienced IDA. Regarding the risk factors of the IDA, this study found that the child’s age, presence of diarrhea or fever, place of residence, household size, wealth status, and access to sanitation facilities are significantly associated with IDA among CU5 in Pakistan. For WRA, this study found education, body mass index, vitamin A and zinc status, household food security status, wealth status, and access to sanitation facilities are significantly associated with IDA. Large, well-established, government-funded programmes focused on micronutrient supplementation, food fortification, diversification of food supplies, and treatment and prevention of infectious and parasitic diseases are needed to prevent IDA and all forms of anemia among children and women in Pakistan.

## Figures and Tables

**Table 1:** Child, maternal, household, and community characteristics of CU5 from the NNS 2018 (*n* = 17,814).

Maternal, Child, and Household Characteristics	*n* (%)
Mother’s Education	
None	10,299 (54.8)
Primary	1990 (12.4)
Middle	1659 (9.7)
Secondary	2073 (12.7)
Higher	1793 (10.4)
Maternal Working Status	
Housewife	15,748 (89.6)
Others	2066 (10.4)
Mother’s Age	
Less than 20 years	238 (1.3)
20–34 years	12,343 (71.5)
35–49 years	5233 (27.2)
**Child Characteristics**	
Gender	
Male	9145 (51.0)
Female	8669 (49.0)
Child’s Age	
6–23 months	3471 (19.4)
24–59 months	14,343 (80.6)
Diarrhea in Last 2 Weeks	
Yes	1504 (7.6)
No	16,310 (92.4)
ARI in Last 2 Weeks	
Yes	486 (1.9)
No	17,328 (98.1)
Fever in Last 2 Weeks	
Yes	2582 (13.7)
No	15,232 (86.3)
Biomarker Results	
Hb, Mean ± SD	10.6 ± 2.5
Ferritin, Mean ± SD	20.2 ± 32.3
IDA	
Deficient (Anemia and Low Ferritin)	4694 (28.9)
Non-Deficient	13,120 (71.1)
**Household Characteristics**	
Family Size	
≤6 members	9884 (57.7)
7 or more members	7930 (42.3)
Number of Children under five	
<5	17,652 (99.1)
≥5	162 (0.9)
Drinking Water Sources	
Improved sources	16,005 (92.4)
Unimproved sources	1809 (7.6)
Sanitation Facilities	
Improved sanitation facility	14,353 (84.4)
Unimproved sanitation facility	3461 (15.6)
Food Insecurity Status	
Food secure	10,787 (60.8)
Mild food insecure	2016 (11.8)
Moderate food insecure	1388 (8.3)
Severe food insecure	3623 (19.1)
Household received Financial Assistance in Last 12 months	
Yes	842 (4.9)
No	16,972 (95.1)
Wealth Status (quintiles)	
Poorest	4380 (19.4)
Second	4144 (20.6)
Middle	3748 (21.1)
Fourth	3251 (21.4)
Richest	2291 (17.5)
**Community Characteristics**	
Area	
Urban	5540 (37.9)
Rural	12,274 (62.1)
Province	
Punjab	7208 (55.1)
Sindh	3532 (27.2)
KP	2086 (9.3)
Balochistan	2272 (4.7)
ICT	178 (0.8)
FATA	302 (0.9)
AJK	1270 (1.6)
GB	966 (0.5)

Abbreviations: ARI, acute respiratory infection; Hb, hemoglobin; SD, standard deviation; IDA, iron-deficiency anemia; KP, Khyber Pakhtunkhwa; ICT, Islamabad Capital Territory; FATA, erstwhile Federally Administered Tribal Areas; AJK, Azad Jammu and Kashmir; GB, Gilgit Baltistan.

**Table 2:** Women’s household and community characteristics of WRA (15–49 years) from the NNS 2018 (*n* = 22,114).

Women’s Household and Community Characteristics	*n* (%)
Women Education	
None	12,664 (53.7)
Primary	2402 (12.1)
Middle	2012 (9.6)
Secondary	2600 (12.9)
Higher	2436 (11.7)
Working Status	
Housewife	18,774 (86.5)
Others	3340 (13.5)
Age	
15–29 years	9605 (44.9)
30–49 years	12,509 (55.1)
BMI (kg/m^2^)	
Underweight (<18.5)	2364 (10.4)
Normal (18.5–24.9)	10,595 (45.4)
Overweight (25.0–29.9)	5853 (27.5)
Obese (≥30)	3302 (16.8)
Marital Status	
Currently Married	20,505 (93.1)
Ever Married	324 (1.6)
Un-Married	1285 (5.3)
Micronutrient Status	
Hb, Mean ± SD	12.1 ± 2.1
Ferritin, Mean ± SD	32.5 ± 61.1
IDA	
Deficient (Anemia and Low Ferritin)	3992 (18.4)
Non-Deficient	18,122 (81.6)
Vitamin A	
Severe (<0.35 µmol/L)	1113 (4.8)
Mild (0.35–0.70 µmol/L)	5083 (22.3)
Non-Deficient (>0.70 µmol/L)	15,918 (72.9)
Zinc	
Deficient (<60 µg/dL)	4809 (22.2)
Non-Deficient (≥60 µg/dL)	17,305 (77.8)
Vitamin D	
Severe Deficiency (<8.0 ng/mL)	6005 (25.2)
Deficiency (8.0–20.0 ng/mL)	11,661 (54.4)
Desirable (>20.0–30.0 ng/mL)	2324 (11.6)
Sufficient (>30.0 ng/mL)	2124 (8.7)
**Household Characteristics**	
Family Size	
≤6 members	12,267 (58.0)
7 or more members	9847 (42.0)
Drinking Water Sources	
Improved sources	19,836 (92.4)
Unimproved sources	2278 (7.6)
Sanitation Facilities	
Improved sanitation facility	17,904 (84.8)
Unimproved sanitation facility	4210 (15.2)
Food Insecurity Status	
Food secure	13,217 (60.3)
Mild food insecure	2607 (11.8)
Moderate food insecure	1723 (8.3)
Severe food insecure	4567 (19.6)
Household received Financial Assistance in Last 12 Months	
Yes	1136 (5.2)
No	20,978 (94.8)
Wealth Status (quintiles)	
Poorest	5427 (18.7)
Second	4964 (19.4)
Middle	4601 (20.9)
Fourth	4047 (21.7)
Richest	3075 (19.2)
**Community Characteristics**	
Area	
Urban	7050 (39.7)
Rural	15,064 (60.3)
Province	
Punjab	8486 (53.7)
Sindh	4495 (28.2)
KP	2568 (9.2)
Balochistan	3048 (4.8)
ICT	285 (1.1)
FATA	394 (0.9)
AJK	1612 (1.7)
GB	1226 (0.5)

Abbreviations: BMI, body mass index; Hb, hemoglobin; SD, standard deviation; IDA, iron-deficiency anemia; KP, Khyber Pakhtunkhwa; ICT, Islamabad Capital Territory; FATA, erstwhile Federally Administered Tribal Areas; AJK, Azad Jammu and Kashmir; GB, Gilgit Baltistan.

**Table 3:** Risk factors of IDA among CU5 in the NNS 2018 (*n* = 17,814).

	Deficient (Anemia and Low Ferritin)	Non-Deficient	Unadjusted Odd Ratio (OR) [95% CI]	*p*-Values	Adjusted Odd Ratio (OR) [95% CI]	*p*-Values
**Maternal Characteristics**						
Mother’s Education						
None	2716 (29.4)	7583 (70.6)	1.203 (1.034–1.4)	0.017		
Primary	568 (31.2)	1422 (68.8)	1.305 (1.085–1.571)	0.005		
Middle	440 (28.9)	1219 (71.1)	1.171 (0.962–1.425)	0.115		
Secondary	543 (26.7)	1530 (73.3)	1.05 (0.872–1.265)	0.607		
Higher	427 (25.7)	1366 (74.3)	Ref.			
Maternal Working Status						
Housewife	4175 (28.8)	11,573 (71.2)	Ref.			
Others	519 (29.5)	1547 (70.5)	1.035 (0.904–1.185)	0.617		
Mother’s Age						
Less than 20 years	61 (26.5)	177 (73.5)	Ref.			
20–34 years	3335 (29.7)	9008 (70.3)	1.173 (0.831–1.656)	0.365		
35–49 years	1298 (26.6)	3935 (73.4)	1.006 (0.709–1.428)	0.974		
**Child Characteristics**						
Gender						
Male	2403 (29.3)	6742 (70.7)	1.047 (0.962–1.139)	0.290		
Female	2291 (28.4)	6378 (71.6)	Ref.			
Child’s Age						
6–23 months	1017 (31.6)	2454 (68.4)	1.179 (1.062–1.31)	0.002	1.198 (1.076–1.333)	0.001
24–59 months	3677 (28.2)	10,666 (71.8)	Ref.		Ref.	
Diarrhea in Last 2 Weeks						
Yes	484 (35.8)	1020 (64.2)	1.415 (1.224–1.637)	<0.001	1.319 (1.127–1.544)	0.001
No	4210 (28.3)	12,100 (71.7)	Ref.		Ref.	
ARI in Last 2 Weeks						
Yes	128 (25.9)	358 (74.1)	0.858 (0.662–1.113)	0.250		
No	4566 (28.9)	12,762 (71.1)	Ref.			
Fever in Last 2 Weeks						
Yes	761 (33.1)	1821 (66.9)	1.263 (1.124–1.421)	<0.001	1.16 (1.023–1.315)	0.02
No	3933 (28.2)	11,299 (71.8)	Ref.		Ref.	
**Household Characteristics**						
Family Size						
≤6 members	2581 (28.0)	7303 (72.0)	Ref.			
7 or more members	2113 (30.1)	5817 (69.9)	1.109 (1.018–1.207)	0.017		
Number of Children under Five						
<5	4639 (28.7)	13,013 (71.3)	Ref.		Ref.	
≥5	55 (42.9)	107 (57.1)	1.867 (1.21–2.879)	0.005	1.997 (1.282–3.11)	0.002
Drinking Water Sources						
Improved sources	4290 (29.2)	11,715 (70.8)	Ref.			
Unimproved sources	404 (24.8)	1405 (75.2)	0.801 (0.679–0.945)	0.009		
Sanitation Facilities						
Improved sanitation facility	3723 (28.0)	10,630 (72.0)	Ref.		Ref.	
Unimproved sanitation facility	971 (33.8)	2490 (66.2)	1.318 (1.184–1.467)	<0.001	1.168 (1.019–1.338)	0.026
Food Insecurity Status						
Food Secure	2782 (28.1)	8005 (71.9)	Ref.		Ref.	
Mild food insecure	508 (28.1)	1508 (71.9)	0.999 (0.87–1.145)	0.983		
Moderate food insecure	415 (32.3)	973 (67.7)	1.22 (1.043–1.427)	0.013		
Severe food insecure	989 (30.3)	2634 (69.7)	1.114 (0.999–1.243)	0.052		
Household received Financial Assistance in Last 12 Months						
Yes	270 (37.6)	572 (62.4)	Ref.		Ref.	
No	4424 (28.4)	12,548 (71.6)	0.66 (0.551–0.792)	<0.001	0.776 (0.641–0.939)	0.009
Wealth Status (quintiles)						
Poorest	1194 (32.7)	3186 (67.3)	1.314 (1.14–1.514)	<0.001	1.271 (1.076–1.502)	0.005
Second	1086 (29.9)	3058 (70.1)	1.15 (0.996–1.327)	0.057	1.222 (1.053–1.418)	0.008
Middle	982 (27.7)	2766 (72.3)	1.034 (0.894–1.195)	0.651	1.088 (0.939–1.259)	0.262
Fourth	835 (27.1)	2416 (72.9)	1.001 (0.863–1.161)	0.994	1.026 (0.884–1.19)	0.738
Richest	597 (27.0)	1694 (73.0)	Ref.		Ref.	
**Community Characteristics**						
Area						
Urban	1477 (28.4)	4063 (71.6)	Ref.			
Rural	3217 (29.2)	9057 (70.8)	1.041 (0.95–1.139)	0.389		
Province						
Punjab	2149 (30.1)	5059 (69.9)	2.487 (2.018–3.064)	<0.001	2.929 (2.365–3.628)	<0.001
Sindh	1170 (32.4)	2362 (67.6)	2.774 (2.236–3.441)	<0.001	2.904 (2.33–3.619)	<0.001
KP	369 (19.8)	1717 (80.2)	1.427 (1.123–1.815)	0.004	1.551 (1.216–1.977)	<0.001
Balochistan	512 (20.9)	1760 (79.1)	1.53 (1.196–1.957)	0.001	1.58 (1.23–2.029)	<0.001
ICT	30 (16.7)	148 (83.3)	1.162 (0.736–1.835)	0.518	1.421 (0.896–2.256)	0.136
FATA	62 (22.0)	240 (78.0)	1.634 (1.106–2.414)	0.014	1.621 (1.092–2.406)	0.017
AJK	246 (18.4)	1024 (81.6)	1.307 (1.002–1.705)	0.048	1.441 (1.099–1.889)	0.008
GB	156 (14.7)	810 (85.3)	Ref.		Ref.	

Abbreviations: CI; confidence interval; ARI, acute respiratory infection; KP, Khyber Pakhtunkhwa; ICT, Islamabad Capital Territory; FATA, erstwhile Federally Administered Tribal Areas; AJK, Azad Jammu and Kashmir; GB, Gilgit Baltistan.

**Table 4:** Risk factors of IDA among WRA in the NNS 2018 (*n* = 22,114).

	Deficient (Anemia and Low Ferritin)	Non-Deficient	Unadjusted Odd Ratio (OR) [95% CI]	*p*-Values	Adjusted Odd Ratio (OR) [95% CI]	*p*-Values
**Women Characteristics**						
Education						
None	2480 (20.7)	10,184 (79.3)	1.708 (1.453–2.006)	<0.001	1.536 (1.3–1.815)	<0.001
Primary	415 (17.1)	1987 (82.9)	1.35 (1.108–1.644)	0.003	1.263 (1.034–1.544)	0.022
Middle	354 (18.0)	1658 (82.0)	1.433 (1.161–1.768)	0.001	1.399 (1.129–1.733)	0.002
Secondary	410 (14.5)	2190 (85.5)	1.106 (0.901–1.358)	0.337	1.086 (0.882–1.338)	0.435
Higher	333 (13.3)	2103 (86.7)	Ref.		Ref.	
Working Status						
Housewife	3351 (18.1)	15,423 (81.9)	Ref.			
Others	641 (19.9)	2699 (80.1)	1.125 (0.996–1.272)	0.058		
Age						
15–29 years	1754 (18.5)	7851 (81.5)	Ref.			
30–49 years	2238 (18.2)	10,271 (81.8)	0.981 (0.899–1.071)	0.668		
BMI (kg/m^2^)						
Underweight (<18.5)	560 (24.3)	1804 (75.7)	1.262 (1.104–1.441)	0.001	1.098 (0.957–1.261)	0.182
Normal (18.5–24.9)	2044 (20.3)	8551 (79.7)	Ref.		Ref.	
Overweight (25.0–29.9)	919 (15.4)	4934 (84.6)	0.714 (0.641–0.796)	<0.001	0.772 (0.691–0.862)	<0.001
Obese (≥30)	469 (14.2)	2833 (85.8)	0.65 (0.567–0.745)	<0.001	0.712 (0.62–0.817)	<0.001
Marital Status						
Currently Married	3714 (18.3)	16,791 (81.7)	1.034 (0.843–1.267)	0.751		
Ever Married	56 (21.2)	268 (78.8)	1.242 (0.82–1.884)	0.306		
Un-Married	222 (17.8)	1063 (82.2)	Ref.			
**Micronutrient Status**						
Vitamin A						
Severe (<0.35 µmol/L)	219 (21.6)	894 (78.4)	1.358 (1.126–1.637)	0.001	1.266 (1.051–1.524)	0.013
Mild (0.35–0.70 µmol/L)	1111 (22.6)	3972 (77.4)	1.44 (1.305–1.589)	<0.001	1.369 (1.238–1.513)	<0.001
Non deficient (>0.70 µmol/L)	2662 (16.8)	13,256 (83.2)	Ref.		Ref.	
Zinc						
Deficient (<60 µg/dL)	1077 (23.4)	3732 (76.6)	1.504 (1.364–1.659)	<0.001	1.424 (1.289–1.573)	<0.001
Non-Deficient (≥60 µg/dL)	2915 (16.9)	14,390 (83.1)	Ref.		Ref.	
Vitamin D						
Severe Deficiency (<8.0 ng/mL)	998 (16.5)	5007 (83.5)	0.961 (0.808–1.143)	0.656		
Deficiency (8.0–20.0 ng/mL)	2173 (18.8)	9488 (81.2)	1.13 (0.966–1.322)	0.125		
Desirable (>20.0–30.0 ng/mL)	469 (21.3)	1855 (78.7)	1.316 (1.09–1.589)	0.004		
Sufficient (>30.0 ng/mL)	352 (17.0)	1772 (83.0)	Ref.			
**Household Characteristics**						
Family size						
≤6 members	2195 (18.1)	10,072 (81.9)	Ref.			
≥7 members	1797 (18.7)	8050 (81.3)	1.043 (0.955–1.138)	0.348		
Drinking Water Sources						
Improved sources	3585 (18.3)	16,251 (81.7)	Ref.			
Unimproved sources	407 (18.6)	1871 (81.4)	1.019 (0.86–1.208)	0.824		
Sanitation Facilities						
Improved sanitation facility	3049 (17.3)	14,855 (82.7)	Ref.			
Unimproved sanitation facility	943 (24.5)	3267 (75.5)	1.557 (1.398–1.733)	<0.001		
Food Insecurity Status						
Food Secure	2149 (16.4)	11,068 (83.6)	Ref.		Ref.	
Mild food insecure	493 (20.2)	2114 (79.8)	1.292 (1.127–1.482)	<0.001	1.223 (1.064–1.405)	0.005
Moderate food insecure	350 (20.6)	1373 (79.4)	1.32 (1.124–1.55)	0.001	1.134 (0.96–1.339)	0.138
Severe food insecure	1000 (22.4)	3567 (77.6)	1.468 (1.319–1.634)	<0.001	1.202 (1.074–1.344)	0.001
Household received Financial Assistance in Last 12 Months						
Yes	247 (25.0)	889 (75.0)	Ref.			
No	3745 (18.0)	17,233 (82.0)	0.657 (0.551–0.784)	<0.001		
Wealth Status (quintiles)						
Poorest	1200 (24.6)	4227 (75.4)	1.753 (1.519–2.025)	<0.001		
Second	920 (19.8)	4044 (80.2)	1.325 (1.143–1.536)	<0.001		
Middle	758 (16.5)	3843 (83.5)	1.058 (0.91–1.231)	0.461		
Fourth	638 (16.0)	3409 (84.0)	1.023 (0.877–1.194)	0.774		
Richest	476 (15.7)	2599 (84.3)	Ref.			
**Community Characteristics**						
Area						
Urban	1236 (17.2)	5814 (82.8)	Ref.			
Rural	2756 (19.1)	12,308 (80.9)	1.133 (1.033–1.243)	0.008		
Province						
Punjab	1522 (17.9)	6964 (82.1)	1.03 (0.854–1.242)	0.758	1.022 (0.842–1.239)	0.828
Sindh	1068 (22.8)	3427 (77.2)	1.396 (1.151–1.692)	0.001	1.25 (1.024–1.526)	0.028
KP	243 (8.9)	2325 (91.1)	0.461 (0.365–0.583)	<0.001	0.443 (0.349–0.562)	<0.001
Balochistan	598 (17.8)	2450 (82.2)	1.023 (0.824–1.27)	0.837	0.873 (0.698–1.092)	0.234
ICT	35 (11.8)	250 (88.2)	0.628 (0.413–0.954)	0.029	0.714 (0.466–1.094)	0.122
FATA	40 (13.6)	354 (86.4)	0.744 (0.467–1.185)	0.213	0.701 (0.44–1.118)	0.136
AJK	273 (16.9)	1339 (83.1)	0.962 (0.751–1.232)	0.757	1.015 (0.789–1.307)	0.906
GB	213 (17.5)	1013 (82.5)	Ref.		Ref.	

Abbreviations: CI; confidence interval; BMI, body mass index; KP, Khyber Pakhtunkhwa; ICT, Islamabad Capital Territory; FATA, erstwhile Federally Administered Tribal Areas; AJK, Azad Jammu and Kashmir; GB, Gilgit Baltistan.

## Data Availability

Data can be requested from the Government of Pakistan with information on specific details regarding planned analyses and outputs.
